# Characterization of Seven Shiga Toxin Phages Induced from Human-Derived Shiga Toxin-Producing *Escherichia coli*

**DOI:** 10.3390/microorganisms13040783

**Published:** 2025-03-28

**Authors:** Xinxia Sui, Shuyun Wang, Xi Yang, Peihua Zhang, Hui Sun, Xiangning Bai, Yanwen Xiong

**Affiliations:** 1 National Institute for Communicable Disease Control and Prevention, Chinese Center for Disease Control and Prevention, Beijing 102206, China; xinxia_sui@163.com (X.S.);; 2 Clinical Laboratory, Children’s Hospital Affiliated to Shandong University, Jinan 250022, China; 3 Department of Microbiology, Division of Laboratory Medicine, Oslo University Hospital, 0372 Oslo, Norway; 4 Hebei Key Laboratory of Intractable Pathogens, Shijiazhuang Center for Disease Control and Prevention, Shijiazhuang 050011, China

**Keywords:** *Escherichia coli*, Shiga toxin (Stx), Stx phages, phage morphology, phage genome

## Abstract

Shiga toxin-producing *Escherichia coli* (STEC) is an important pathogen that can cause asymptomatic infections, diarrhea, hemorrhagic colitis (HC), and life-threatening hemolytic uremic syndrome (HUS) in humans. Shiga toxins (Stxs) are the major virulence factors encoded by prophages, which play a crucial role in STEC pathogenesis and evolution. In this study, seven Stx phages were obtained from STEC isolates derived from four asymptomatic food handlers, two diarrheal patients, and one outbreak-related HUS case in China. These phages exhibited three morphologies: an icosahedral head with either a short or a long tail, and an elongated head with a long tail. Of these seven phages, three were sequenced; two showed a complete identity with their respective prophage sequences, while phage phiXuzhou21-Stx2a lacked a 6011 bp region-encoding integrase, excisionase, and hypothetical proteins. Comparative genome analysis revealed that the induced seven phages primarily varied in their regulatory regions, whereas the short-tailed phages showed high similarity in their morphogenesis-related regions. In addition, five of the seven phages demonstrated the ability to convert non-pathogenic *E. coli* strains into Stx-producing transduced strains. Under inducing conditions, Stx expression levels were significantly increased in these transduced strains. These findings underscore the diversity and adaptability of Stx phages and emphasize the importance of understanding their genetic and molecular interactions with host bacteria.

## 1. Introduction

Shiga toxin-producing *Escherichia coli* (STEC) is a foodborne zoonotic pathogen, which causes a spectrum of illnesses, from asymptomatic infections to mild or severe diarrhea, and in some cases, it can lead to more serious conditions, such as hemorrhagic colitis (HC) and hemolytic uremic syndrome (HUS) [1]. It has been estimated that STEC causes 2.8 million acute infections worldwide each year, resulting in 3890 cases of HUS and 230 deaths [1]. STEC may lead to human illness through the fecal-oral route of contaminated food, exposure to contaminated irrigation water, and direct contact with animals [2].

Shiga toxin (Stx) is a key virulence factor in STEC strains, exhibiting cytotoxic effects on target cells [3]. STEC can produce two types of Stx: Stx1 and Stx2, which can be further divided into various subtypes, with three for Stx1 (Stx1a, Stx1c, and Stx1d), and at least seven for Stx2 (Stx2a to Stx2g). Recently, additional Stx subtypes have been designated, ranging from Stx2h to Stx2o [4]. The Stx subtypes have been associated with different levels of virulence, and Stx2 is more frequently associated with HC and HUS than Stx1 [5,6].

Shiga toxin is encoded by *stx* genes within the genome of a prophage located in the bacterial chromosome. This type of prophage is referred to as a Stx prophage. When STEC strains are exposed to various external stresses, the SOS response is triggered. This leads to the release of Stx phages. These free phage particles have the potential to convert non-pathogenic *E. coli* strains into STEC [7]. The spreading of Stx phages might contribute to the emergence of hybrids of STEC pathogens, such as the *E. coli* O104:H4 strain [8]. This strain caused a total of 3816 infections and 54 deaths in Germany and is characterized as enteroaggregative *E. coli* (EAEC) that acquired an Stx2a phage. STEC hybrids, including those with enterotoxigenic properties, have been reported to be associated with HUS in humans [9,10,11].

Previous studies have identified Stx phages from strains associated with outbreaks and diarrheal patients, such as phages VT2-Sa, 933W, and 2851 [12,13,14], but the phages from isolates derived from asymptomatic carriers remain underexplored. In some countries, STEC infection is a notifiable disease, and asymptomatic carriers are legally prohibited from working in food processing [15,16]. Stx phage particles can retain their infectivity in food and under food processing conditions [17], emphasizing the potentials of asymptomatic carriers in the transmission dynamics of STEC. In this study, we aimed to comprehensively characterize Stx phages induced from STEC strains originating from three distinct sources: asymptomatic food handlers, diarrheal patients, and an outbreak-related HUS case. Our primary objective was to investigate whether there are discernible differences in the morphology, genetic diversity, and transfection properties of Stx phages across these different host populations, thereby gaining deeper insights into the factors that contribute to the varying clinical outcomes associated with STEC infections.

## 2. Materials and Methods

### 2.1. Bacterial Strains

In this study, seven human-derived STEC strains from our previous investigations were selected. These included four strains (STEC1586, STEC1588, STEC1589, and STEC1590) from asymptomatic food handlers in Guangxi [18], two strains (STEC799 and STEC801) from diarrheal patients in Beijing, and one strain (Xuzhou21) from an HUS case. Strain Xuzhou21 was isolated from a patient with HUS during the massive O157:H7 outbreak in 1999 in Xuzhou, China, which led to 195 hospitalizations for HUS and resulted in 177 fatalities [19]. The complete genomes of these seven STEC strains are available in NCBI with the accession numbers SAMN36269289, SAMN36269387, SAMN36269400, SAMN36269620, SAMN44480233, SAMN44480234, and SAMN44480235.

### 2.2. Induction, Isolation, and Purification of Stx Phages

The strains were inoculated into Luria-Bertani (LB) broth and incubated overnight at 37 °C with shaking (220 rpm). The cultures were then diluted 1:100 into fresh LB broth containing 5 mM CaCl_2_ and incubated at 37 °C with shaking. When the culture reached an OD_600_ of 0.5, mitomycin C (Solarbio, Beijing, China) was added to a final concentration of 0.5 μg/mL. After incubation for 12 h at 37 °C with shaking in the dark, the culture was centrifuged at 4000× *g* for 10 min to separate the bacterial cells and debris from the phage particles. The supernatant was filtered through a 0.22 μm filter, and the filtrate served as the phage suspension.

The isolation of Stx phages was performed using the double-layer agar plate method, with *E. coli* strains MG1655 and C600 serving as host strains for the phage screening. Two hundred microliters of each phage suspension were mixed with an equal volume of log-phase host strain and incubated for 30 min at 37 °C. This mixture was added to 5 mL molten LB soft agar (top agar), poured over LB agar plates, and allowed to solidify. After incubation for 18 h at 37 °C, the plates were examined for plaque formation. Individual plaques were randomly selected and screened by PCR using *stx*-specific primers [20]. Positive plaques were purified through three consecutive rounds of plaque purification [21].

### 2.3. Phage Enrichment and DNA Extraction

Ten milliliters (10 mL) of purified phage (10^4^ PFU/mL) was added to 200 mL of log-phase host culture and incubated overnight at 37 °C with shaking. The culture was then centrifuged, and the supernatant was filtered through a 0.22 μm filter. DNase I (1 mg/mL) (Solarbio, Beijing, China) and RNase A (1 mg/mL) (CWBIO, Beijing, China) were added to the filtrate and incubated at 37 °C for 1 h. Then, 1 M NaCl was added, and the mixture was placed on ice for 1 h. After centrifugation at 10,000× *g* for 30 min, 10% (*w*/*v*) polyethylene glycol 8000 (PEG 8000) was added to the supernatant and incubated overnight at 4 °C. After centrifugation at 8000× *g* for 1 h, the precipitate was resuspended in SM buffer [0.58% NaCl, 0.2% MgSO_4_·7H_2_O, 50 mM Tris–HCl (pH 7.5), and 0.01% gelatin] (Coolaber, Beijing, China) and extracted twice with chloroform.

Each 600 μL of phage suspension was treated with 0.6 μL of DNase I (1 mg/mL) and 0.6 μL of RNase A (1 mg/mL), and incubated at 37 °C overnight to remove free DNA and RNA. Next, the phage suspensions were treated with 10 mg/mL Proteinase K (Solarbio, Beijing, China) and 10% SDS for 1 h to disrupt the phage capsid. Finally, the DNA was extracted using the phenol-chloroform method [22].

### 2.4. Phage Genome Sequencing, Assembly, and Annotation

The phage genomes were sequenced on the MGISEQ-2000 platform (MGI Tech Co., Ltd., Shenzhen, China) with 150 bp paired-end reads. Low-quality reads, defined as those with consecutive bases covered by fewer than five reads, and adapter sequences were removed using the SOAPnuke software (v1.5.6) [23]. The filtered raw sequencing reads were then assembled using Unicycler (v0.5.1) [24].

Coding sequences (CDSs) were identified and annotated using the RAST annotation server (https://rast.nmpdr.org/, accessed on 10 April 2024). Nucleotide sequences were aligned using BLASTn from the NCBI suite (https://blast.ncbi.nlm.nih.gov, accessed on 10 April 2024). The tRNAs in the genome were predicted using tRNAscan-SE 2.0 [25]. Prophages within bacterial genome sequences were predicted and annotated using PHASTER (http://phaster.ca/, accessed on 15 March 2024) [26]. The gene adjacent to the integrase was identified as the phage insertion site [27]. Nucleotide intergenomic similarity (NIS) values were calculated using the online tool VIRIDIC (http://rhea.icbm.uni-oldenburg.de/VIRIDIC/, accessed on 20 May 2024) [28]. Phages are considered the same species if their nucleotide sequences exhibit more than 95% identity across their entire genomes in pairwise comparisons. Phages are classified into the same genus if they share over 70% nucleotide identity [29].

### 2.5. Comparative Analysis of Stx Prophage and Stx Phages

The sequence of the Stx phage and its prophage were compared to assess genomic alterations during prophage induction. Stx prophage sequences were predicted from the complete bacterial genomes of *E. coli* O157:H7 strains Xuzhou21 and STEC801 and *E. coli* O112:H8 strain STEC1589, using PHASTER. The resulting Stx prophage sequences were then used to compare with the Stx phages (phiXuzhou21-Stx2a, phiSTEC801-Stx1a, and phiSTEC1589-Stx1c) induced from the same host strains. The comparative genomics were visualized using Easyfig_2.2.5 [30].

### 2.6. Transmission Electron Microscopy of Purified Phages

Ten microliters (10 μL) of purified phages were placed on carbon-coated Formvar films for 10 min, followed by staining with 3% phosphotungstic acid for 2 min. Excess stain was absorbed using filter paper. The phage morphology was examined using transmission electron microscopy (JEM-1200EX, Jeol Ltd., Tokyo Japan) operated at 100 kV [31].

### 2.7. Stx Phage Transduction

To analyze the host range of seven Stx phages, the non-pathogenic *E. coli* strains (MC1061 and MG1655) and pathogenic *E. coli* strains were chosen as bacterial hosts. The pathogenic *E. coli* strains include four categories of diarrheagenic *E. coli* strains.

Five hundred microliters (500 μL) of each phage suspension were mixed with an equal amount of logarithmic phase host strain and incubated overnight at 37 °C. The mixtures were subjected to a ten-fold serial dilution in PBS. One hundred microliters (100 μL) of each dilution were plated onto LB plates. After incubation at 37 °C for 24 h, several colonies from each plate were randomly selected for PCR with *stx*-specific primers [20] to identify positive colonies indicative of lysogenic bacteria. Positive colonies confirmed as lysogenic bacteria were subcultured three times on LB agar to examine the stability of the lysogeny.

### 2.8. RNA Extraction and Relative Quantification of Stx Transcription

The parental STEC strains and Stx transduced strains were selected for *stx1*/*stx2* transcriptional expression analysis. Overnight cultures were transferred to fresh LB medium and incubated at 37 °C with shaking until OD_600_ reached approximately 0.6. Each culture was then divided into two tubes, with mitomycin C added to one of the tubes at a final concentration of 0.5 µg/mL. All cultures were incubated at 37 °C with shaking for 3 h. Total RNA was extracted using the RNeasy Mini Kit (Qiagen, Hilden, Germany). To address potential gDNA contamination, especially given the low abundance of *stx* mRNA, we used the RNase-Free DNase Set (Qiagen, Hilden, Germany) with additional steps to remove genomic DNA, according to the manufacturer’s instructions. Reverse-quantitative PCR (RT-qPCR) was performed on a Rotor-Gene Q Real-Time PCR system (Qiagen, Hilden, Germany) using the HiScript^®^ II One Step qRT-PCR SYBR^®^ Green Kit (Vazyme, Nanjing, China), according to the manufacturer’s instructions. The relative expression levels of *stx* genes were normalized to an endogenous reference gene (*gapA*). The Stx expression levels in mitomycin C-induced cells relative to non-induced ones were calculated. Data were calculated based on ΔCt = Ct*_stx_* − Ct*_gapA_* and ΔΔCt = ΔCt_induced_ − ΔCt_control_. Fold change was calculated by the 2^−ΔΔCt^ method. For real-time PCR, the following primer pairs were used: *stx1* (5′-GGAATTTACCTTAGAYTTCTCRAC-3′ and 5′-CCTGTGCCACTATCAATCATC-3′), *stx2* (5′-TCCATGACAACGGACAGCAG-3′ and 5′-ACGCCAGATATGATGAAACCAG-3′), and the housekeeping gene *gapA* (5′-TATGACTGGTCCGTCTAAAGACAA-3′ and 5′-GGTTTTCTGAGTAGCGGTAGTAGC-3′) [18].

### 2.9. Statistical Analysis

Statistical analysis was performed using GraphPad Prism 9. Student’s *t*-test (two-tailed) was used to compare Stx expression levels between the parental strains and the transduced strains. Statistically significant results were defined as *p*-values ≤ 0.05.

## 3. Results

### 3.1. Characterization of the Stx Prophages and Induced Stx Phages

Among the seven STEC strains, five (Xuzhou21, STEC801, STEC1586, STEC1588, and STEC1590) carried both Stx1 and Stx2 prophages. Strains STEC799 and STEC1589 harbored only Stx2 and Stx1 prophage, respectively. The genome sizes of these 12 prophages ranged from 45,120 bp to 63,085 bp. Seven insertion sites were identified, including *yehV* (encoding an HTH-type transcriptional regulator MlrA), *wrbA* (encoding a NAD(P)H: quinone oxidoreductase), *sbcB* (encoding an exodeoxyribonuclease I), *tRNA-Arg*, *dmsB* (encoding a dimethyl sulfoxide reductase subunit B), *YfhL* (encoding a putative 4Fe-4S cluster-containing protein YfhL), and *dusA* (encoding a tRNA dihydroxyuridine synthase A), respectively. The insertion site of the Stx1a prophage in strain STEC1588 remained undetermined (Table 1).

Among the seven STEC strains carrying one or two Stx prophages, only one phage was successfully induced and isolated from each strain. VIRIDIC analysis revealed that the phages phiXuzhou21-Stx2a and phiSTEC801-Stx1a belong to the genus *Traversvirus;* the phage phiSTEC799-Stx2c belongs to the genus *Pankowvirus*; the phages phiSTEC1586-Stx1c, phiSTEC1589-Stx1c, and phiSTEC1590-Stx1c belong to the genus *Diegovirus.* The phage phiSTEC1588-Stx2a could not be determined to any known genus (Table 1).

### 3.2. Morphology of Seven Stx Phages

**Figure 1:**
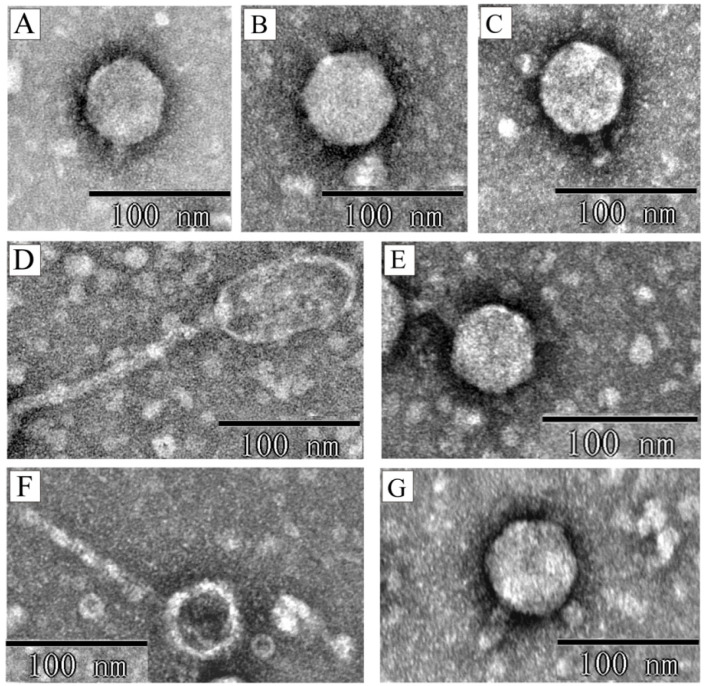
The transmission electron microscopy of seven Stx phage particles. Electron micrographs depict the following phages: (**A**) phiXuzhou21-Stx2a; (**B**) phiSTEC801-Stx1a; (**C**) phiSTEC1586-Stx1c; (**D**) phiSTEC799-Stx2c; (**E**) phiSTEC1589-Stx1c; (**F**) phiSTEC1588-Stx2a; (**G**) phiSTEC1590-Stx1c. Five morphologies observed: an icosahedral head with a short tail (**A**–**C**,**E**,**G**), an elongated head with a long tail (**D**), and an icosahedral head with a long tail (**F**).

The seven phages exhibited three distinct morphologies. Five phages (phiXuzhou21-Stx2a, phiSTEC801-Stx1a, phiSTEC1586-Stx1c, phiSTEC1589-Stx1c, and phiSTEC1590-Stx1c) displayed an icosahedral head and a short tail. The head diameters ranged from 51 nm to 65 nm, and the tail lengths ranged from 12 nm to 32 nm (Figure 1A–C,E,G). Phage phiSTEC799-Stx2c presented a prolate head measuring approximately 43 nm in width and approximately 86 nm in length, with a long, non-contractile tail of approximately 155 nm (Figure 1D). Phage phiSTEC1588-Stx2a had an icosahedral head with a diameter of approximately 38 nm and a non-contractile tail approximately 119 nm in length (Figure 1F).

### 3.3. Genomic Comparison Between the Stx Phage and Its Prophage

To investigate potential nucleotide sequence alterations during prophage induction, the genomes of three phages (phiXuzhou21-Stx2a, phiSTEC801-Stx1a, and phiSTEC1589-Stx1c) were sequenced and compared to their corresponding prophage sequences. Blastn analyses revealed that phages phiSTEC801-Stx1a and phiSTEC1589-Stx1c maintained 100% nucleotide sequence identity with their respective prophage sequences.

**Figure 2:**
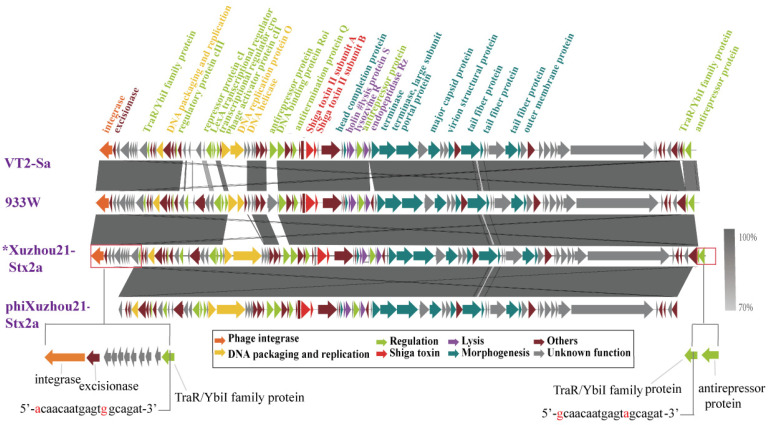
Comparison of Stx2a phage genomes. Sequence of phage phiXuzhou21-Stx2a obtained in this study was compared with the sequences of phages VT2-Sa and 933W. The genetic map was constructed using Easyfig. Each arrow indicates predicted ORF, whereas the color of the arrow represents predicted functional proteins. The gray shadows represent the nucleotide identity between the two phages. * indicates a Stx2a prophage sequence. The red-framed section highlights genomic differences between phage phiXuzhou21-Stx2a and its prophage. Two 19 bp direct-repeat sequences at the termini of phage phiXuzhou21-Stx2a are shown at the bottom of the figure.

However, the genome of phiXuzhou21-Stx2a showed 91% coverage compared to its prophage sequence. The phage genome was 56,998 bp in length, while the prophage sequence was 63,009 bp, resulting in a 6011 bp deletion. In the upstream region of the prophage sequence (positions 1–4844 bp), 10 CDSs encoding integrase, excisionase, and hypothetical proteins were absent in the phage genome. Additionally, in the downstream region of the prophage sequence (positions 62,111–62,738 bp), a CDS encoding the phage antirepressor Ant was absent in the phage genome. The sequences of the other regions between the phage and its prophage differed by only one nucleotide. At the two termini, a 19 bp direct-repeat sequence with two nucleotide differences was present (Figure 2).

To further investigate the sequence variations between the induced Stx phages and their prophages, a comparative analysis was conducted on phiXuzhou21-2a and two other phages, VT2-Sa and 933W. These two Stx2a phages were induced from the O157:H7 outbreak-related strains Sakai and EDL933, respectively. The genomes of phages VT2-Sa and 933W were identical to their corresponding prophage sequences. In comparison with the genomes of phages VT2-Sa and 933W, phiXuzhou21-Stx2a showed high similarity in the toxin/lysis and morphology module regions, while the regulatory region exhibited significant variability. Notably, phiXuzhou21-Stx2a also featured a 6011 bp deletion (Figure 2).

### 3.4. Genomic Comparison Among the Seven Stx Phage/Prophages

**Figure 3:**
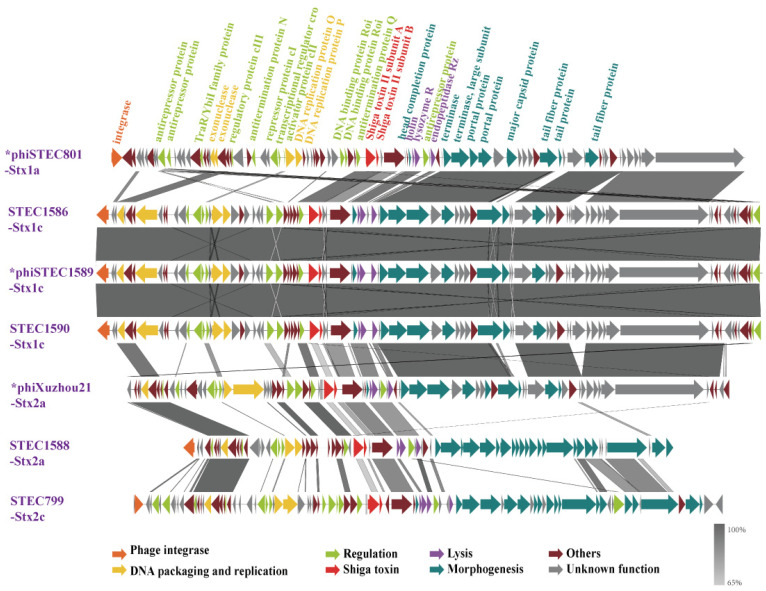
Genomic structure and nucleotide sequence comparison of seven Stx phages/prophages in this study. The genetic map was constructed using Easyfig. Each arrow represents a predicted open reading frame (ORF), with the color of the arrow indicating the predicted functional protein. Gray shading highlights regions of nucleotide identity between the two phages. An asterisk (*) denotes induced and sequenced Stx phage genomes, while the others represent prophage genomes. The five phages in the above section of the figure are short-tailed phages, with genome variations predominantly found in the replication and regulatory regions. In contrast, the two phages in the below section of the figure are long-tailed phages, which share only a few homologous nucleotide sequences with the short-tailed phages.

A comparative genomics analysis was performed to evaluate the genomic sequence features among the seven phages. The phage genomes consisted of three primary segments: a DNA packaging/replication and regulation module, a toxin/lysis module, and a morphology module. Apart from the three Stx1c phages, which showed considerable sequence conservation, significant sequence variation was observed, particularly in the replication and regulation module (Figure 3). Among the five short-tailed phage genomes, variations were predominantly noted in the replication and regulatory region. And the genes encoding morphogenesis proteins showed high similarity, including those for the phage head completion protein, portal proteins, and major capsid proteins.

The two long-tailed phages, phiSTEC799-Stx2c and phiSTEC1588-Stx2a, displayed substantial differences from the others. Only a few replication and regulatory proteins, along with four tail morphogenesis proteins, including three tail tip assembly proteins and one tail tip host specificity protein, were homologous between these two phages and the others. Notably, despite encoding the same Stx2a subtype, two phages—phiXuzhou21-Stx2a and phiSTEC1588-Stx2a—exhibited significant genomic and morphological differences (Figure 3).

### 3.5. Properties of Stx Phage Transduction

The ability of Stx phages to infect and lysogenize different susceptible bacterial strains was assessed. *E. coli* strain MG1655 was successfully lysogenized by five phages: phiSTEC799-Stx2c, phiSTEC1586-Stx1c, phiSTEC1588-Stx2a, phiSTEC1589-Stx1c, and phiSTEC1590-Stx1c. *E. coli* strain MC1061 was efficiently lysogenized by three phages: phiSTEC1586-Stx1c, phiSTEC1589-Stx1c, and phiSTEC1590-Stx1c. The transduced strains remained stable after three successive subcultures. Strains MG1655 and MC1061 could be infected by the phage phiXuzhou21-Stx2a; however, the resulting transduced strains were unstable following sub-cultivation. Phage phiSTEC801-Stx1a was not observed to form lysogens. Compared to non-pathogenic *E. coli*, all seven Stx phages were unable to transduce other pathogenic *E. coli* strains (Supplementary Table S1).

### 3.6. Variable Stx mRNA Expression Levels in Host Strains

**Figure 4:**
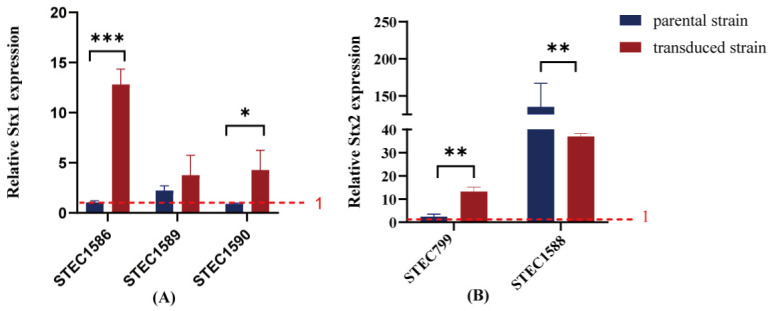
Fold change in *stx* mRNA transcription was compared between transduced strains and their original strains. Stx expression above the red dashed line (set to 1) indicates that Stx expression levels increase after mitomycin C induction, while Stx expression below the red dashed line suggests that Stx expression levels remain unchanged following mitomycin C induction. Data are present as means ± standard deviations from three independent replicates. (**A**) Fold change in *stx1* mRNA transcription; (**B**) fold change in *stx2* mRNA transcription. */**/*** indicates statistically significant differences.

The Stx transcription levels in five transduced strains were evaluated. Stx expression in all transduced strains was inducible by mitomycin C. Compared to their corresponding parental strains, two out of three Stx1-transduced strains exhibited higher levels of *stx1* mRNA transcription following mitomycin C induction (Figure 4A). For the two Stx2-transduced strains, one Stx2c-producing strain showed higher inducibility than its parental strain, whereas the other Stx2a-producing strain demonstrated less inducibility despite maintaining a high level of Stx2 expression (Figure 4B).

## 4. Discussion

Stx phages are capable of transferring between different *E. coli* strains through horizontal gene transfer, thereby playing a crucial role in the spread of Stx and contributing to the emergence of new pathogenic strains. Previous studies have identified Stx phages from strains associated with outbreaks and diarrheal patients, but the exploration of phages from strains derived from asymptomatic carriers has been limited. In this study, seven Stx phages were obtained from seven STEC isolates derived from four asymptomatic food handlers, two diarrheal patients, and one outbreak-related HUS case in China. Among the five STEC strains carrying two Stx prophages, only one phage was successfully induced and isolated from each strain. One reason for the failure to produce a phage is the incomplete genome. Specifically, the Stx2b prophages in both STEC1586 and STEC1590 lack certain critical regions (e.g., the regulatory region), rendering them potentially defective. However, in the other three strains containing two prophages, the genomes are predicted to be complete. The inability to isolate these prophages may be due to within-host competition among multiple prophages or concentrations too low to detect effectively [32]. According to the latest taxonomic guidelines, six of the seven phages belong to three genera: *Diegovirus*, *Traversvirus*, and *Pankowvirus.* Phage phiSTEC1588-Stx2a, however, based on its nucleotide identity score (NIS < 70%), was not assigned to any existing genus and may represent a novel genus within the class *Caudoviricetes*. The identification of a potentially novel genus further emphasizes the need for continued research into the taxonomy, ecology, and functional roles of Stx phages.

The integrase gene plays a pivotal role in the lysogenic cycle of temperate phages. Prior studies have demonstrated that the genetic diversity of integrase correlates with the variability in prophage insertion site [33]. In this study, seven distinct insertion sites were identified among 12 prophages. Notably, the predicted Stx1a prophage was integrated into the *yehV* site, while the Stx2a prophage was inserted into the *wrbA* site within the genome of strain Xuzhou21, a pattern similar to that observed in strains Sakai and EDL933 [12,13]. Several studies have investigated the occupancy of integration sites in *E. coli* O157 strains by Stx prophages. These studies indicate that the *yehV* site is the most frequently occupied, followed by *wrbA*, *argW*, and *sbcB* [34]. The *sbcB* site was also detected in *E. coli* O157 strains STEC799 and STEC801. Additionally, a new insertion site, *dmsB*, was identified in three strains derived from asymptomatic carriers, highlighting the diversity of integration sites. This diversity in integration sites underscores the adaptability and potential for horizontal gene transfer among Stx phages.

Stx phages exhibit a common head-tail structure, characterized by icosahedral or elongated heads and either contractile or non-contractile long tails or short tails [35]. In this study, the seven Stx phages showed variability in their morphology, with three distinct shapes observed. Five phages displayed hexagonal heads and short tails, a morphology similar to many Stx phages isolated from *E. coli* strains such as Sakai, EDL933, and CB13374 [12,13,36], and also observed in Stx phages from *Shigella* strains [37]. This type of morphology is prevalent, with approximately 70% of induced Stx phages having icosahedral heads and short tails [38]. The other two phages were long-tailed. PhiSTEC1588-Stx2a, an Stx2a phage, possessed a hexagonal head and a long tail, resembling the previously isolated Stx2k phage [39]. PhiSTEC799-Stx2c, an Stx2c phage, displayed an elongated head and a long tail, similar to the previously described Stx1 phage H19J [40]. These two long-tailed phages have also been isolated from STEC strains of different origins in other countries [41,42]. These observations confirm the diversity of phage morphologies circulating within the STEC population. The presence of both short-tailed and long-tailed phages highlights the adaptability of Stx phages to different ecological niches and hosts.

Of the seven phages, three were sequenced and compared with their corresponding prophage sequences. The analysis revealed that two phage genomes were completely identical to the prophage counterparts. This finding aligns with previous studies demonstrating high nucleotide similarity between induced Stx phages and prophage sequences of both human and environmental origin [39,43]. However, the genome of phage phiXuzhou-Stx2a exhibited differences from its prophage genomes. Notably, it lacked genes encoding integrase, excisionase, and some other proteins. These findings contrast with those observed in Stx2a phages VT2-Sa and 933W, which showed complete identity with their corresponding prophage sequences. We found that 10 CDSs were absent in the phage phiXuzhou21-Stx2a genome compared to its prophage genome and observed that phiXuzhou21-Stx2a transduced strains were unstable following sub-cultivation. The integrase and excisionase play critical roles in mediating the integration and excision of the phages into and from the host genome [44], which may affect the phage’s ability to infect and establish stable lysogeny. We hypothesize that phiXuzhou-Stx2a may not integrate its genome into the bacterial chromosome due to the absence of these key elements, and instead, it might exist in a carrier state [45].

A comparative genome analysis was conducted on the seven phages or prophages. The five short-tailed phage genomes primarily exhibited variations in their regulatory regions. A previous study analyzed 11 Stx2 phages associated with human STEC infections worldwide. Except for one phage, all others harbored integrase and key regulatory elements characteristic of lambdoid phages. Notably, nucleotide sequence variation among Stx2 phages is particularly pronounced within the regulatory regions [46]. Pinto et al. analyzed 279 prophage and phage genomes retrieved from GenBank and concluded that the structural diversity in lysis-lysogeny regulatory regions is significant [47]. Atitkar et al. found that the mosaicism of Stx phage regulatory sequences, along with potential differences in CI function, influences downstream Stx production and could be used to predict the virulence of STEC clinical isolates [48]. Our analysis revealed that the genomic variations in the five short-tailed phages are primarily concentrated in their regulatory regions, consistent with these studies. In contrast to the variability observed in regulatory regions, high homology was observed in genes encoding key phage morphogenesis proteins, including major capsid proteins, portal proteins, structural proteins, and tail proteins. This pattern is consistent with findings from other studies [43], indicating that these conserved genes play critical roles in phage structure and assembly. The short-tailed Stx phages demonstrated greater genomic relatedness, likely driven by their shared host range, which facilitates appreciable levels of genomic recombination [46].

In contrast to the short-tailed phages, the two long-tailed phages, phiSTEC799-Stx2c and phiSTEC1588-Stx2a, exhibited greater genomic variation. Despite this diversity, these long-tailed phages showed higher similarity in the genes encoding three tail tip assembly proteins and one tail tip host specificity protein. The tail tip host specificity protein is involved in the phage attachment to the host receptor and the induction of phage DNA ejection, distinguishing it from the mechanism used by short-tailed phages, which rely on phage tail fiber proteins for host recognition [43].

Lysogenic phage transduction is an efficient mechanism for the rapid spread of phage-encoded virulence genes [49]. In this study, *E. coli* strains MG1655 and MC1061 were efficiently lysogenized or infected by most Stx phages, forming stable lysogens. However, these two host strains could be infected by the phage phiXuzhou21-Stx2a, but the transduced strains were unstable after sub-cultivation. Previous reports have shown that Stx phages can infect non-pathogenic *E. coli* strains and form new lysogens, which may appear to be carried transiently [49,50]. This instability suggests that phiXuzhou21-Stx2a may not integrate efficiently into the bacterial chromosome or lacks key factors necessary for stable maintenance within the host. A variety of heterotypic pathogenic *E. coli* strains has been identified, indicating that Stx phages possess the ability to transfer *stx* genes among different pathotypes of *E. coli* strains [50]. Although phage-mediated lysogen of pathogenic *E. coli* was not observed in this study, this does not imply that the phages lacked infectivity. Several factors may contribute to this inability, including the lack of the correct receptor on the bacterial surface required for phage attachment [50]. Moreover, when a temperate phage integrates into the host bacterial genome, it induces superinfection exclusion. Consequently, strains that harbor the temperate phage genome in their chromosomes become resistant to subsequent infections by the same phage and phages sharing the same immunity region [51]. Further research into the genetic and molecular factors influencing the interactions between Stx phages and their bacterial hosts is crucial for elucidating the mechanisms of horizontal gene transfer and the evolution of pathogenicity. By targeting the mechanisms through which Stx phages are released from host bacteria and subsequently infect other cells, it might be possible to develop strategies to mitigate the dissemination of Shiga toxin, thereby offering a new approach to managing STEC infection.

Under inducing conditions, Stx expression levels were increased in the five transduced strains formed in *E. coli* MG1655. Notably, the highest mRNA transcription of *stx* was observed in the transduced strain of phiSTEC1588-Stx2a carrying Stx2a, which was related to the severe clinical outcome [52]. The two Stx1-producing and one Stx2-producing transduced strains also exhibited higher level of *stx* mRNA transcription compared to their corresponding parental strains. Previous studies have demonstrated that newly converted strains harboring Stx phages are significantly more virulent than non-converted strains *in vivo* experiments [50,53]. The variability in Stx expression among different transduced strains suggests that both the genetic makeup of the phage and the host bacterium play crucial roles in determining the level of toxin production.

In conclusion, the seven Stx phages of human-derived STEC strains exhibited significant diversity in terms of their insertion site, morphology, taxonomy, and genomic composition. The identification of novel integration sites and the variability in phage morphology indicate that our knowledge of Stx phages remains incomplete. The ability of Stx phages to integrate into diverse bacterial genomes and induce high levels of toxin expression in newly converted strains suggests their potentials of transmission among strains and pathogenicity. A broader array of samples would enable us to more comprehensively explore the variability in Stx phage properties.

## Figures and Tables

**Table 1:** Characterization of the Stx prophages and induced Stx phages.

Strains	Stx Prophages			Stx Phages			
	Stx Subtype	Insertion Site	Positions on the Chromosomes (bp)	Phage Name	Taxonomy (Genus)	Lysogenization	Accession Number
Xuzhou21	Stx1a *	*yehV*	1726316–1773296	NA	NA	NA	NA
(O157:H7)	Stx2a	*wrbA*	3287084–3349824	phiXuzhou21-Stx2a	*Traversvirus*	MG1655, MC1061	PQ600366
STEC799	Stx2c	*sbcB*	1932228–1987965	phiSTEC799-Stx2c	*Pankowvirus*	MG1655	-
(O157:H7)							
STEC801	Stx1a	*tRNA-Arg*	1486902–1546771	phiSTEC801-Stx1a	*Traversvirus*	Not	PQ600365
(O157:H7)	Stx2c *	*sbcB*	1979419–2034452	NA	NA	NA	NA
STEC1586	Stx1c	*dmsB*	1199974–1262789	phiSTEC1586-Stx1c	*Diegovirus*	MG1655, MC1061	-
(O112:H8)	Stx2b *	*yfhL*	31350–77421	NA	NA	NA	NA
STEC1588	Stx1a *	unknown	3162659–3209046	NA	NA	NA	NA
(O112:H19)	Stx2a	*dusA*	4416471–4461590	phiSTEC1588-Stx2a	unknown	MG1655	-
STEC1589	Stx1c	*dmsB*	2427601–2490685	phiSTEC1589-Stx1c	*Diegovirus*	MG1655, MC1061	PQ600364
(O112:H8)							
STEC1590	Stx1c	*dmsB*	2476392–2539212	phiSTEC1590-Stx1c	*Diegovirus*	MG1655, MC1061	-
(O112:H8)	Stx2b *	*yfhL*	1307496–1355384	NA	NA	NA	NA

“*” The induction and isolation of Stx phages failed. “-” genomes of induced Stx phages are not sequenced. NA, not applicable.

## Data Availability

The original contributions presented in the study are included in the article (Table 1), further inquiries can be directed to the corresponding author.

## Supplementary Materials

The following supporting information can be downloaded at: https://www.mdpi.com/article/10.3390/microorganisms13040783/s1, Table S1: Transduction of the seven Stx phages.
