# Supplementary material for: Characterization of Seven Shiga Toxin Phages Induced from Human-Derived Shiga Toxin-Producing Escherichia coli
Source: Microorganisms. 2025 Mar 28;13(4):783. doi: 10.3390/microorganisms13040783 (PMC12029490; doi:10.3390/microorganisms13040783)
Supplement: Supplementary file 1 [file microorganisms-13-00783-s001.zip › microorganisms-3422893-supplementary.pdf]

Supplementary Table S1 Transduction of the seven Stx phages.

Pathogenic types	Recipient strain	Stx phages						
		phiXuzhou21-Stx2a	phiSTEC799-Stx2c	phiSTEC801-Stx1a	phiSTEC1586-Stx1c	phiSTEC1588-Stx2a	phiSTEC1589-Stx1c	phiSTEC1590-Stx1c
Non-pathogenic *E. coli*	MC1061	+*	-	-	+	-	+	+
	MG1655	+*	+	-	+	+	+	+
EAEC	Ec042	-	-	-	-	-	-	-
	EA194	-	-	-	-	-	-	-
EPEC	E2348/69	-	-	-	-	-	-	-
	EP037	-	-	-	-	-	-	-
	EP185	-	-	-	-	-	-	-
	EP487	-	-	-	-	-	-	-
	EP539	-	-	-	-	-	-	-
ETEC	10407	-	-	-	-	-	-	-
EIEC	44825	-	-	-	-	-	-	-

+ and − indicate positive and negative of *stx* genes, respectively.

*It indicates the transduced strains were unstable after sub-cultivating.
